# Editorial for the Special Issue on Nanogenerators in Korea

**DOI:** 10.3390/mi10020097

**Published:** 2019-01-29

**Authors:** Yong Tae Park, Dukhyun Choi

**Affiliations:** 1 Department of Mechanical Engineering, Myongji University, Yongin, Gyeonggi 17058, Korea; 2 Department of Mechanical Engineering, Kyung Hee University, Yongin, Gyeonggi 17104, Korea

Nanogenerator-based technologies have found outstanding accomplishments in energy harvesting applications over the past two decades. These new power production systems include thermoelectric, piezoelectric, and triboelectric nanogenerators, which have great advantages such as eco-friendly low-cost materials, simple fabrication methods, and operability with various input sources. Since their introduction, many novel designs and applications of nanogenerators as power suppliers and physical sensors have been demonstrated based on their unique advantages. This Special Issue in Micromachines, titled “Nanogenerators in Korea”, compiles some of the recent research accomplishments in the field of nanogenerators for energy harvesting. It consists of 12 papers, which cover both the fundamentals and applications of nanogenerators, including two review papers. These papers can be categorized into four groups as follows:

(1) Triboelectric Nanogenerators (TENG). Lee et al. [1] provided an educational review of PVDF-based triboelectric energy harvesters and self-powered sensors. PVDF is a promising dielectric material for energy harvesting due to its interesting multi-faceted properties, which can be further improved through composites. Kang et al. [2] studied energy harvesting from suspension systems of vehicles. Such an energy harvester could support the ADAS technology in autonomous vehicles. Hwang et al. [3] investigated a gapless structure triboelectric nanogenerator using a mesoporous and deformable Al_2_O_3_–PDMS composite. They also studied its pressure sensitivity and showed its application in smart cushions for monitoring human sitting positions. Lee et al. [4] proposed a spherical TENG structure that utilized both solid–solid contact and liquid–solid contact for water wave energy harvesting. The innovative hybrid design could scavenge greater amounts of energy than the individual methods used separately. Chung et al. [5] investigated an easy-to-fabricate water–solid contact TENG, the surface of which was made superhydrophobic by a simple spray-on technique. The electrical output could be maximized by maintaining a Cassie–Baxter state between the water and the superhydrophobic surface. La et al. [6] proposed a metal-to-metal imprinting process to create micro- and nano-scale structures on the surface of aluminum, which formed one of the layers of the TENG. The nano-structured aluminum showed enhanced output compared to non-structured aluminum. Park et al. [7] investigated the effect of embedding highly dielectric TiO_2_ nanoparticles in PDMS to improve the TENG performance. They also demonstrated the output enhancement using a windmill-integrated TENG system.
(2) Thermoelectric Nanogenerators. Culebras et al. [8] provided a comprehensive review of organic thermoelectric materials and their corresponding composites, with a focus on polymers and carbon nanofillers. Strategies to enhance the thermoelectric performance, polymer composite-based thermoelectric devices, and brief conclusions and outlooks for future research were summarized. Ahn et al. [9] designed an optimized thermoelectric energy harvesting system and applied it on a rolling stock as low-power sensor nodes in a self-powered independent monitoring system. Jang et al. [10] investigated the thermoelectric performance of carbonaceous nanomaterials-based polymeric multilayer structures, showing p-type or n-type thermoelectric properties by simply changing the electrolyte.
(3) Piezoelectric Nanogenerators. Shin et al. [11] investigated the fatigue resistance of piezoelectric PVDF by subjecting the device to 10^7^ cycles of tension and compression. The tension experiments showed stable polarization, while the compression experiments showed a 7% decrease in polarization. However, no notable decrease in output voltage was observed.
(4) Metamaterial Nanogenerators. Lee et al. [12] investigated energy-harvesting metamaterials for a novel wireless-powered chemical sensing system. The resonance frequency and voltage output from the metamaterial changed depending on the chemical compound and its concentration in the channel.

We would like to thank all the authors for their papers submitted to this Special Issue. We would also like to acknowledge all the reviewers for their careful and timely reviews to help improve the quality of this Special Issue.
